# Supplementary material for: A high-resolution mRNA expression time course of embryonic development in zebrafish
Source: eLife. 2017 Nov 16;6:e30860. doi: 10.7554/eLife.30860 (PMC5690287; doi:10.7554/eLife.30860)
Supplement: Supplementary file 6. [file elife-30860-supp6.zip › biolayout-clusters-files/Cluster186-genes.html]

Cluster186


# Cluster186: Genes

| Ensembl ID | Gene Name | Chr | Start | End | Biotype |
| --- | --- | --- | --- | --- | --- |
| ENSDARG00000014039 | ENSDARG00000014039 | 20 | 42640658 | 42664630 | protein\_coding |
| ENSDARG00000078481 | OGFOD3 | 12 | 28218377 | 28234174 | protein\_coding |
| ENSDARG00000051809 | btr12 | 7 | 73448024 | 73463742 | protein\_coding |
| ENSDARG00000104574 | pcsk6 | 7 | 8833689 | 9031807 | protein\_coding |
| ENSDARG00000021677 | phf11 | 9 | 29806108 | 29823608 | protein\_coding |
| ENSDARG00000040005 | scfd2 | 20 | 22900184 | 23132611 | protein\_coding |
| ENSDARG00000093978 | zgc:77486 | 8 | 19222788 | 19235996 | protein\_coding |
